# Factors Associated with Medication Adherence for People Living with Acute HIV Infection in a Tertiary Chinese Hospital in Beijing

**DOI:** 10.1155/2021/1078716

**Published:** 2021-01-05

**Authors:** Xiaolan Wang, Dongmei Li, Meixia Gao, Yuefang Zhou, Caiping Guo, Tong Zhang, Lili Zhang, Wen Wang

**Affiliations:** ^1^Center for Infectious Diseases, Beijing Youan Hospital, Capital Medical University, Beijing 100069, China; ^2^Department of Nursing, Beijing Youan Hospital, Capital Medical University, Beijing 100069, China

## Abstract

Both the management and caregiving intervention of people living with HIV (PLWH), especially during acute HIV-1 infection, represent a public health issue and a form of social support. This current study analyzed the demographic and clinical factors associated with antiretroviral therapy (ART) adherence of PLWH from positive HIV diagnosis to ART initiation in a tertiary Chinese hospital in Beijing. A total of 200 participants diagnosed with acute HIV-1 infection were enrolled in this study. We collected demographic and clinical data by the use of a self-reported questionnaire. Bivariate and multivariate logistic regressions were used to determine associations between potential variables and outcomes. We found that medication adherence was impacted by years of ART and number of reminders (all *P* < 0.05). In addition, medication adherence was associated with viral load at 48 weeks (*P* = 0.035). Future studies are needed to investigate effective interventions that could facilitate ART adherence.

## 1. Introduction

The total number of people living with HIV (PLWH) and acquired immunodeficiency syndrome (AIDS) was 831 225 by the end of July 2018 in China [1]. The number of men who have sex with men (MSM) infected with HIV continues to increase in China [2–6]. In China, rapid upward trends occurred in the proportion of HIV-1-infected MSM, the percentages of which increased by 7.3%, 12.2%, 29.4%, and 25.8% in 2005, 2007, 2011, and 2014, respectively, suggesting that the MSM now constitute the population in which HIV-1 transmission is rapidly increasing [4, 6–9]. There is a large number of MSM in Beijing, one of the most MSM-friendly cities in China, although the spread of HIV/AIDS through sexual contact also increased from 87.1% in 2011 to 96.9% at the end of 2016 in this city [10]. Acute HIV-1 infection is associated with a rapid rise in HIV plasma viremia and commonly manifested with clinically reported symptoms of fever, headache, etc. [11]. Definitions of acute, early, or chronic infection have been demonstrated using the evolution of HIV plasma viremia and HIV-specific antibody [12–15]. Acute and early HIV-1 infection is a major contributor to the epidemic spread of HIV and limiting this spread through “test and treat” strategies may require treatment of persons during the acute phase of infection [11, 16, 17]. Intervention during this stage of infection can dramatically reduce HIV transmission [12, 18–20]. Therefore, the management and care of PLWH, especially during acute HIV-1 infection, are both a public health and social issue. It is very important to understand the demographic and clinical factors affecting the increased HIV epidemic of MSM in Beijing and to promote HIV testing, prevention counseling, and other health care and social services that could reduce HIV and STI transmission.

Antiretroviral therapy (ART) has been shown to be effective in reducing viral loads in PLWH, virus infectivity, and both morbidity and mortality of AIDS-related diseases [21, 22]. In the effort to end the HIV/AIDS epidemic by 2030, the World Health Organization (WHO) updated the ART guidelines in 2015, and all PLWH worldwide were recommended to receive ART as early as possible, regardless of CD4 T-cell count or WHO clinical stage [21]. In 2017, of the 37.9 million PLWH, 24.5 million had access to ART, representing an important milestone in the fight against HIV/AIDS; however, there remains a large gap between the reported percentage and 90% adherence, as the estimated adherence ranges from 55.7% to 85.9% in China [21, 23–25]. China's National Free Antiretroviral Treatment Program (NFATP) was initiated as a pilot in 2003, and enormous efforts have been made to improve the capability to treat the large number of PLWH in China [26, 27]. However, adherence to ART is a complex behavior that is influenced by many demographic and clinical factors, including availability of reminders, substance use, dietary diversity, CD4 T-cell count, depressive symptoms, adverse effects of ART, duration of ART, etc. [28–33]. Thus, the prevention of PLWH from loss to follow-up and the maintenance of optimal ART adherence remain major challenges in China [34–36].

Despite increasing evidence of the importance of engagement in care, little is known about the characteristics of people living with HIV acute infection and factors associated with their antiretroviral medication adherence. Here, in this study, we analyzed the medication adherence of 200 people living with acute HIV infection in a tertiary Chinese hospital in Beijing. This study is aimed at providing empirical evidence for interventions for people living with acute HIV infection.

## 2. Methods

### 2.1. Study Subjects

In this study, participants were enrolled from the Beijing PRIMO cohort or were living with acute HIV infection but were not included in the PRIMO cohort since the initiation of the main cohort. The PRIMO cohort is a prospective clinical cohort of uninfected MSM to identify acute HIV infection at Beijing Youan Hospital, a tertiary Chinese hospital in Beijing, China [9]. After enrollment, the participants were screened for plasma HIV-1 antibody, HIV-1 RNA level, and clinical signs of acute infection, syphilis, and HBV/HCV coinfection status. They were then followed up every 2 months, and HIV-1 antibody was detected at each visit. AHI was defined as a negative or indeterminate HIV-1 antibody status but a positive HIV RNA status [37]. Moreover, people living with acute HIV were identified and then treated under early ART with tenofovir disoproxil fumarate (TDF), lamivudine (3TC), and efavirenz (EFV), all of which are recommended as a first-line regimen in China. In addition, their health status was monitored at each visit at 1, 2, 4, 8, and 12 weeks and every 3 months thereafter. Acute/early HIV-1 infection was classified according to the Fiebig stages based on the characteristics of PLWH [15]. At baseline, 200 eligible participants were identified at Fiebig stage III-VI. Participants were at least 20 years of age, received ART one year after the diagnosis of HIV acute infection, provided written informed consent, voluntarily participated in this study, and were capable of clear awareness and normal expression. Excluded participants were those who suffered from cognitive disorder, oral expression deficiency, mental diseases, or presented with other severe chronic diseases.

### 2.2. Measurements of Independent Variables and Outcomes

All independent variables, including age, level of education, employment, ART reminders, source of assistance, first closure target, years of ART, mental health and marital status, were self-reported by all included participants. All independent variables were categorized as two or three kinds to satisfy the requirement of statistical power. Medication adherence was defined as a dichotomized outcome (more than one pill missed or no pill missed in the previous month).

### 2.3. Statistical Analysis

We generated sample characteristics by performing a descriptive analysis and assessed bivariable associations between our outcome variable (medication adherence) and potential predictors using logistic regression tests. Variables significant in bivariable analysis (*P* < 0.05) were incorporated into a multivariable logistic regression model. In addition, we also compared the CD4 and viral suppression rates at each time point between the missed and unmissed groups. All analyses were conducted by SPSS version 22.0 (IBM Corp. 2013).

## 3. Results

### 3.1. General Characteristics of the Study Participants

In this study, at baseline, 200 eligible PLWH were ultimately selected and enrolled in the intervention group. Two hundred questionnaires were distributed and collected, with a response rate of 100%. All PLWH were infected by unprotected sexual behavior. The majority of PLWH were between 30 and 50 years old, with a median age of 33.07 years. PLWH were mostly single, with a high school or below level of education, were self-reported staff workers, and were migrants from other cities (Table 1).

### 3.2. Factors Associated with Medication Adherence

Among all the participants, 17.2% missed ART. The final multivariable model suggested that participants who were subjected to ART for more than three years had significantly higher odds of missed medication (Table 2). Conversely, using more reminders had significantly lower odds of missed medication.

### 3.3. Association between Medication Adherence, CD4 T-Cell Count, and Viral Load

No between-group differences in CD4 T-cell counts at any point were observed. However, between-group differences in viral suppression rate at 48 weeks were detected (*P* = 0.035). The detailed information is provided in Table 3.

## 4. Discussion

Current ART has transformed HIV/AIDS from an acute, often lethal disease to a chronic disease, leading to an average life expectancy comparable to that of healthy persons [38]. However, ART adherence is crucial for the quality of life of PLWH, and this mostly depends on influences from the various sources, including material and mental health support from their family, spouse, partner, or friends [29, 30, 39]. Hence, it is very important to investigate the social support, social care, and strategies of implementation towards this PLWH population [34, 35, 40]. Therefore, we examined the characteristics of PLWH during acute infection and the influence of demographic and clinical factors on ART adherence. To our knowledge, this study is one of the first efforts to investigate the accuracy of ART adherence among people living with acute HIV in China using a large Beijing PRIMO cohort.

Years of ART emerge as one of the significant contributors to the odds of missed medication. Participants experiencing a relatively higher number of years of ART may encounter several concurrent barriers towards medication adherence. The cumulative side effects of ART may be associated with a lower level of perspective memory performance, which is a key to medication adherence [41, 42]. The situation may be getting even worse as participants get older due to the combination pathology of aging and HIV infection [43]. Another barrier that participants may face is hopelessness. Participants may feel that they have lost control of their life and experience low self-efficacy, which may also be related to increased odds of missed medication [44, 45]. Interventions should be tailored to address concurrent side effects of ART and hopelessness.

PLWH should have the option to have more reminders for medication. The study showed statistical significance between the number of reminders and medication adherence, indicating that those using more reminders tend to have lower odds of missed medication. This reflects a compensatory mechanism of cognitive deficit in many HIV-positive participants. Therefore, emphasis should be made to hold discussions with patients to work together to find the most suitable reminder of medication. These patients should be taught practical skills concerning medicine management to avoid missing medication, overcome side effects of medicine, and develop a life habit that includes taking medicine [46]. Interventions aimed at improving medication adherence conducted in older adults living with chronic disease could be transferred to the field of HIV care. For example, a meta-analysis summarizing the effectiveness of the teach-back method found that this method not only can improve adherence but also can positively affect self-efficacy [47]. Other methods, such as patient education (e.g., recurrent and personalized telephone counseling sessions with health educators), medication regimen management (using combinatory pills to reduce the number of pills patients take daily), clinical pharmacist consultation for chronic disease comanagement, cognitive behavioral therapies, medication reminders, and incentives to promote adherence, should be integrated into routine care of HIV participants [48].

In addition, we found that missed medication was associated with a reduced suppression rate at week 48. However, we did not longitudinally explore this association. Therefore, future longitudinal studies are needed to verify the temporal relationship between medication adherence and HIV clinical outcomes.

Several limitations should also be addressed: (1) the nature of the cross-sectional study from which we could not draw temporal relationships between potential variables and outcomes; (2) the small sample size, which could cause selection bias; and 3) the missing variables that limited our ability to draw a more comprehensive picture. Future studies with large sample sizes and multiple waves are needed to address these limitations.

## 5. Conclusions

This study revealed that medication adherence is impacted by various factors, such as years of ART and number of reminders. Future studies are needed to investigate effective interventions that could facilitate ART adherence.

## Figures and Tables

**Table 1:** Baseline characteristics of participants.

	Missed medication	Unmissed medication
Age		
20-30	6 (18.2)	75 (46.3)
30-50	24 (72.7)	81 (50)
50 and more	3 (9.1)	6 (3.7)
Level of education		
High school or below	18 (54.5)	83 (49.7)
College or above	15 (45.5)	84 (50.3)
Employment		
Employed or self-employed	19 (57.6)	120 (76.4)
Unemployed or student	14 (42.4)	37 (23.6)
Marital status		
Married	14 (43.8)	37 (22.6)
Unmarried or widowed or divorced	18 (56.3)	127 (77.4)
Years on ART		
One-three	5 (17.9)	81 (73.6)
Three or more	23 (82.1)	29 (26.4)
Number of reminders		
No	23 (67.6)	8 (4.9)
One	6 (17.6)	135 (83.3)
Two or more	5 (14.7)	19 (11.7)
First disclosure target		
Family members	11 (34.4)	65 (45.1)
Friends or sex partner	11 (34.4)	51 (35.4)
Other	10 (31.3)	28 (19.4)
Number of assistance sources		
No	17 (50)	55 (33.5)
One	12 (35.3)	67 (40.9)
Two or more	5 (14.7)	42 (25.6)
Mental health		
Very good/good	23 (67.6)	110 (68.8)
Very bad/bad/fair	11 (32.4)	50 (31.3)

**Table 2:** Binary and multivariate logistic regression of factors associated with medication adherence.

	Binary analyses			Multivariate analyses		
	OR	95% CI	*P*	OR	95% CI	*P*
Age						
20-30	1					
30-50	3.704	1.435-9.559	0.007	1.79	0.364-8.806	0.474
50 and above	6.25	1.242-31.463	0.026	1.444	0.141-14.838	0.757
Level of education						
High school or below	1					
College or above	0.81	0.539-1.203	0.159			
Employment						
Unemployed	1			1		
Students or employed	0.418	0.191-0.915	0.029	0.508	0.133-1.933	0.32
Marital status						
Married	1			1		
Single, widowed, and divorced	2.67	1.213-5.874	0.015	1.251	0.303-5.16	0.757
Years on ART						
1-3	1			1		
3 years or more	12.848	4.469-36.938	<0.001	10.568	2.781-40.156	<0.001
Number of reminders						
No	1			1		
One	0.015	0.005-0.049	<0.001	0.033	0.008-0.139	<0.001
Two or more	0.092	0.026-0.327	<0.001	0.139	0.028-0.695	0.016
Number of assistance						
No	1					
One	0.579	0.255-1.316	0.192			
Two or more	0.385	0.131-1.128	0.082			
First disclosure target						
Parents/spouse	1					
Friends/sexual partners	1.275	0.512-3.175	0.602			
Others	2.11	0.805-5.535	0.129			
Mental health						
Fair/bad/very bad	1					
Very good/good	0.95	0.43-2.099	0.9			

**Table 3:** Association between medication adherence, CD4 T-cell count, and viral load.

	CD4 T-cell count			Percentage of viral load < 50 copies/ml		
	Mean and standard deviation			Number and percentage		
	0 w	24 w	48 w	0 w	24 w	48 w
Missed	440 (195)	580 (231)	630 (475)	0	33 (97.1)	21 (61.8)
Unmissed	414 (206)	602 (215)	589 (205)	0	148 (92.1)	124 (75.6)
*P*	0.506	0.614	0.628		0.89	0.035

## Data Availability

The dataset used and/or analyzed during the current study is available from the corresponding author on reasonable request.
